# Phenotypic correlations of *CALR* mutation variant allele frequency in patients with myelofibrosis

**DOI:** 10.1038/s41408-023-00786-x

**Published:** 2023-01-30

**Authors:** Paola Guglielmelli, Chiara Maccari, Benedetta Sordi, Manjola Balliu, Alessandro Atanasio, Carmela Mannarelli, Giulio Capecchi, Ilaria Sestini, Giacomo Coltro, Giuseppe Gaetano Loscocco, Giada Rotunno, Eva Angori, Filippo C. Borri, Ayalew Tefferi, Alessandro M. Vannucchi

**Affiliations:** 1 CRIMM, Center Research and Innovation of Myeloproliferative Neoplasms, University of Florence, AOU Careggi, Florence, Italy; 2 Doctorate School GenOMec, University of Siena, Siena, Italy; 3 University of Florence, Florence, Italy; 4 Division of Hematology, Mayo Clinic, Rochester, MN USA

**Keywords:** Myeloproliferative disease, Oncogenesis

Dear Editor,

Somatic mutations in *calreticulin* (*CALR*), encoding for the reticulum-endoplasmic-associated Ca^2+^-binding chaperone protein calreticulin, located at chromosome 19p13.13, represent the second most frequent driver mutation in myeloproliferative neoplasms (MPN) [1, 2]. *CALR* mutation is harbored by 20–30% of patients with essential thrombocythemia (ET) and 25–35% of prefibrotic primary myelofibrosis (pre-PMF) and overt PMF [3]. There are two main types of *CALR* mutation: Type 1, a del52 bp deletion, and similar abnormalities (defined as Type 1-like based on similar structural characteristics), and Type 2, a 5 bp insertion, and Type 2-like mutations, all located in exon 9 and causing a reading frameshift. Most patients have *CALR* variant allele frequency (VAF) below 50% (conventionally defined as heterozygous), as opposite to a few with higher VAF (above 50%, homozygous). Homozygosity, that may be associated with uniparental disomy (UPD)/copy neutral loss-of-heterozigosity (CN-LOH) at chr19 [4], indicates the presence of cells with UPD/CN-LOH in a variable context of heterozygous cells. *CALR* homozygosity is hallmarked by myeloperoxidase (MPO) deficiency in myeloid cells due to abnormal proteosomal degradation of immature protein [5]. In patients with *JAK2*V617F mutation, phenotypic and prognostic correlates of higher vs. lower VAF were reported across the spectrum of MPN [6], and variably included correlation of higher VAF with higher red blood cell values and neutrophil counts, lower platelet counts, larger spleen, pruritus, venous thrombosis [7], and transformation to secondary forms of MF [8]. Remarkably, *JAK2*V617F VAF in the lowest quartile was associated with shorter overall survival (OS) and leukemia-free survival (LFS) in patients with PMF [9, 10]. Conversely, little information is available regarding the phenotypic and/or prognostic impact of *CALR* VAF. The aim of this study was to characterize the hematological and clinical correlates of *CALR* VAF in patients with PMF and post-essential thrombocythemia myelofibrosis (PET-MF).

All patients with confirmed WHO2016 and IWG-MRT diagnosis of PMF and PET-MF, respectively, available at the Center Research and Innovation of Myeloproliferative Neoplasms (CRIMM), Florence, were included in the study (MYNERVA project, IRB approval #14560). *CALR* mutation was assayed by PCR amplification and capillary gel electrophoresis starting from granulocyte DNA. *CALR* VAF was determined by automated interpolation of the area under the curve, and expressed as the ratio between the mutant peak area and the sum of mutant and wild-type peak areas x100. Mutation analysis of a panel of 40 myeloid neoplasm-associated genes, including *CALR*, was performed by NGS (Oncomine, ThermoFisher) [11]. The nonparametric Wilcoxon rank-sum test, Kaplan–Meier estimate of survival and log-rank test were used as appropriate. Reported *P*-values are two-sided; *P* < 0.05 was considered statistically significant.

We first compared the *CALR* VAF values obtained with capillary gel electrophoresis and NGS. The median values of the two methods were comparable (51.0 ± 9.7% vs. 51.1 ± 9.9%). The Spearman’s rho test showed a highly significant correlation (*r* = 0.61; 95% confidence interval [CI] 0.47–0.72, *P* < 0.0001); the bias between the two determinations (Bland–Altman test) was −0.33 (95% CI, −12.8–12.1; Supplemental Fig. 1). Therefore, results from capillary gel electrophoresis were confidently used throughout the study.

A total of 620 patients with MF were analyzed, 481 (77.6%) PMF and 139 (22.4%) PET-MF. Median follow-up was 4.1 years, range 0.3–28.5 years. Mutation distribution for *JAK2*V617F, *MPL*W515 and triple-negativity was 69.2% (*n* = 333), 6.0% (*n* = 29) and 10.0% (*n* = 48) for PMF, and 48.3% (*n* = 73), 11.9% (*n* = 18), and 3.6% (*n* = 5) for PET-MF. One-hundred twenty-one patients were *CALR* mutated, 42 pre-PMF (34.7%), 36 overt PMF (29.8%) and 43 PET-MF (35.5%) (Table 1 and Supplemental Table 1). A Type 1/like mutation occurred in 87 patients (71.9%), 8 (6.6%) were Type 1-like; Type 2/like mutation was found in 34 patients (28.1%), 3 (2.5%) were Type 2-like. The median *CALR* VAF in the entire population was 51.4 ± 10.4%, and respective figures for pre-PMF, overt PMF and PET-MF were 48.9 ± 8.9%, 52.5 ± 11.4%, and 59.9 ± 10.7%. For the purposes of the analysis, we considered as having a high VAF (*CALR*-high) those patients whose *CALR* VAF was ≥95% CI of the distribution of VAFs in the entire population, corresponding to a VAF threshold of ≥55%; patients with *CALR* VAF < 55% were considered as having a low VAF (*CARL*-low).

**Table 1:** Clinical and hematologic characteristics of 121 *CALR*-mutated patients with primary myelofibrosis and post-essential thrombocythemia myelofibrosis (PET-MF) divided according to their VAF ≥ 55% (*CALR*-high) and <55% (*CALR*-low).

Variables	*CALR*-low (*N* = 93)	*CALR*-high (*N* = 28)	*P*
*CALR* mutation type			
Type1/1-like	67 (72.0)	20 (71.4)	0.95
Type2/2-like	26 (28.0)	8 (28.6)	
Follow-up (yrs); median (range)	6.0 (0.3–37.1)	4.8 (0.6–30.1)	0.55
PMF, *n* (%)	64 (68.8)	14 (50.0)	0.07
PET-MF, *n* (%)	29 (31.2)	14 (50.0)	
Males; *n* (%)	51 (54.8)	16 (57.1)	0.83
Age (yrs); median (range)	57.9 (18–84)	60.2 (29–82)	0.30
Age >65 y; *n* (%)	28 (30.1)	6 (21.4)	0.37
White blood cells, x10^9^/L; median (range)	8.1 (2.5–56.9)	7.3 (4.0–36.0)	0.24
Hemoglobin, g/L; median (range)	12.1 (7.4–15.3)	10.8 (8.3–14.6)	**0.02**
Platelets, x10^9^/L; median (range)	661 (57–1800)	446 (102–1053)	**0.02**
Circulating blasts ≥2%; *n* (%)	1.1 ± 2.7	0.7 ± 1.0	0.69
CD34 + x10^6^/L; median (range)	16.9 (0.0–2143)	62.2 (4–3432)	**0.01**
LDH > UNL; *n* (%)(*n* = 85)	58 (86.6)	18 (100)	0.10
Splenomegaly; *n* %)(*n* = 115)	59 (67.8)	20 (71.4)	0.72
Spleen >10 cm from LCM (*n* = 115)	25 (28.7)	12 (42.6)	0.46
Constitutional symptoms; *n* (%)(*n* = 116)	20 (22.5)	10 (37.0)	0.13
Karyotype Information; *n* (%) (*n* = 90)			
Abnormal cytogenetics	11 (16.2)	6 (27.3)	0.25
Unfavorable karyotype^a^	5 (7.4)	3 (13.6)	0.30
IPSS; *n* (%)			
Low	43 (46.2)	10 (35.7)	
Intermediate-1	25 (26.9)	9 (32.1)	0.73
Intermediate-2	14 (15.1)	6 (21.4)	
High	11 (11.8)	3 (10.7)	
Total major thrombosis events; *n* (%)	9 (9.7)	2 (7.1)	0.68
Total major bleeding events; *n* (%)	14 (15.1)	2 (7.4)	0.30
Acute leukemia progression; *n* (%)	7 (7.5)	2 (7.1)	0.95
Death; *n* (%)	31 (33.3)	12 (42.9)	0.36
HMR category; *n* (%)	30 (32.3)	14 (50.0)	0.14
≥2 HMR mutated genes	8 (8.6)	6 (21.4)	0.09
Patients with additional mutated myeloid genes; *n* (%)	50 (57.5)	23 (82.1)	**0.02**

*IPSS* International Prognostic Scoring System, *HMR* high molecular risk category, points to the presence of any one mutation in *ASXL1, EZH2, SRSF2, IDH1/2* and *U2AF1*. *HMR* *≥* *2* means the presence of 2 or more HMR mutated genes; 2 or more mutations in the same gene were counted as one.

^a^Unfavorable karyotype indicates any abnormal karyotype other than normal karyotype or sole abnormalities of 20q-, 13q-, +9, chromosome 1 translocation/duplication, -Y or sex chromosome abnormality other than –Y.

Bold values denote statistical significance at the *p* < 0.05 level.

*CALR*-high was found in 28 cases, accounting for 23.1% of the study population (Table 1). There was a trend to increased representation of *CALR*-high patients from pre-PMF (*n* = 6, 14.3%) to overt PMF (*n* = 8, 22.2%) to PET-MF (*n* = 14, 32.6%). Conversely, Type 1/1-like and Type 2/2-like mutations were similarly represented in the different diagnostic categories and among *CALR*-high and *CALR*-low patients, 72% and 28% and 71% and 29%, respectively. There was no significant difference between *CALR*-high and *CALR*-low patients regarding gender, age, leukocytes count, IPSS score, BM fibrosis grade 3 (58.3% and 44.8%), karyotype abnormalities, constitutional symptoms, splenomegaly, thrombosis and bleeding events. Statistically significant lower Hb level (10.8 g/dl (range, 8.3–14.5) vs. 12.1 (7.4–16); *P* = 0.02) and platelet count (466 × 10^9^/L (102–1053) vs. 661 (57–1800; *P* = 0.02), higher peripheral blood CD34^+^ cell counts (62.2 × 10^6^/L (4–3452) vs. 16.9 (0–2143); *P* = 0.01) and need of cytoreduction-therapy (78.6% vs. 58.1%, *P* = 0.04), were found by comparing *CALR*-high to *CARL*-low patients. High molecular risk mutations (HMR) were found in 44 patients (36.4%), with a trend to be more frequent among *CALR*-high (50% vs. 32%). *ASXL1* mutations were significantly enriched among *CALR*-high patients (46.4% vs. 27.6%; *P* = 0.04) as it was the presence of ≥1 mutated myeloid gene (82.1% vs. 57.5%, *P* = 0.02).

Twenty-seven patients (22.3%) were treated with ruxolitinib, accounting for 18 (19.3%) and 9 (32.1%) of *CALR*-high and -low patients. An IWG-MRT/ELN-defined splenomegaly response was achieved by 39% and 56% of patients respectively (*P* = 0.45), while refractoriness leading to ruxolitinib stop occurred in 57% and 20% (*P* = 0.30).

The median OS was shorter in *CALR*-high patients, 8.7 years (6.6–10.8) vs. 14.8 years (5.6–24.1), although not statistically different (*P* = 0.36); LFS was similar (Fig. 1A, B). As reported, a Type 1/1-like mutation was associated with improved OS compared to Type 2/2-like (23.8 years (12-2–35.3) vs. 8.6 years (7.5–9.5); HR 2.6 (95% CI 1.1–6.3; *P* = 0.02) in PMF only, and irrelevant in PET-MF. Most common cause of death was leukemia (6 and 1 case in *CALR*-high and low, respectively) and disease progression (5 and 4 cases); there were 2 cases of severe infection-related deaths, both in *CALR*-low patients. A greater proportion of *CALR*-high patients developed anemia during the FU and required RBC transfusion support (32.1% vs. 17.4%, *P* = .037). Kaplan–Meyer analysis revealed an anemia-free survival (Hb <10 g/dL) of 7.9 years (5.7–10 y) in *CALR*-high vs. 22.1 years (13.9–30.3 y) in *CALR*-low patients, *P* = 0.004 (Fig. 1C) with HR 3.1 (95%CI, 1.4–7.0). Also leukocytosis-free survival (leukocytes >25 × 10^9^/L) was shorter in *CALR*-high patients (*P* = 0.04), HR 2.55 (95%CI, 1.0–6.6). (Fig. 1D), while there was no difference for thrombocytopenia-free survival (*P* = 0.23).

**Fig. 1:**
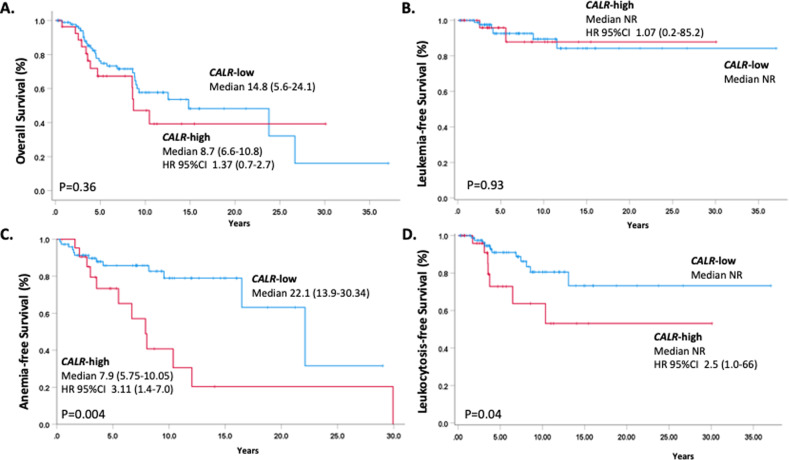
Impact of CALR mutation variant allele frequency on outcomes in myelofibrosis. Kaplan–Meier analysis of Overall Survival (panel **A**), Leukemia-free Survival (panel **B**), Anemia-free Survival (panel **C**) and Leukocytosis-free Survival (panel **D**) in CALR-mutated patients according to their high and low variant allele frequency burden. Please refer to text for definitions and details. HR 95% CI, hazard ratio, 95% confidence Interval.

In this series of 121 *CALR*-mutated patients with myelofibrosis, to our knowledge the largest specifically addressing clinical and hematologic phenotype in relation to mutation VAF, we found 23% of the patients harboring a higher VAF, herein defined as ≥55%. Unlike in patients with *JAK2*V617F mutation, a higher VAF was not associated with striking clinical and hematologic phenotypic differences. Although OS was shorter in the group with higher VAF, the difference did not reach the significance level likely due to the relatively small number of events. Interestingly, we found that a higher VAF was associated with a more anemic phenotype at diagnosis (significantly lower hemoglobin levels) and greater risk of developing anemia in the FU. Together with evidence of elevated CD34^+^ cell counts [12], more *ASXL1* mutated patients [13], greater number of myeloid gene mutations [14], and shorter leukocytosis-free survival, findings support that patients with higher VAF are characterized by a more advanced disease and are at greater risk of progression. Clarification of this point will require larger patient series and longer follow-up.

Conversely, we did not found an impact of *CALR* homozygosity on infectious rate, as it might have been expected by the reported associated deficiency of MPO; indeed, in the original study [5], 2 of 5 patients had severe infections. One possible limitation is that our database included only severe infections, and mild infectious events might have been underrated.

Klampfl et al. observed 3 cases with Type 2 variant and homozygosity, associated with UPD [2]. In 11 cases with *CALR*-high VAF (defined as >60%) and associated UPD/CN-LOH, preferential involvement of Type 2 and rare mutation types was reported [4]. A prevalence of Type 2/2-like mutations was also observed in 5 *CALR* homozygous patients with concurrent MPO deficiency [5]. Conversely, 13 patients with high VAF but without evidence of UPD/CN-LOH were enriched in Type 1, 2 of them showing partial chr19 trisomy. The reasons for higher *CALR* VAF in the absence of UPD/CN-LOH remain unclear. In our series, we did not find differences in the frequency of Type1 and Type2, and atypical/like, mutations in *CALR*-high vs. *CALR*-low patients, but we did not evaluate CN-LOH as an underlying genetic mechanism. A correlation of CN-LOH and accelerated phase was also reported [4]. An unique case of a patient with concomitant *CALR* Type1 and *BCR::ABL1* mutation was described, where *CALR* homozygosity originated from the founding heterozygous clone that later acquired, in an independent branch, the *BCR::ABL1* abnormality [15].

In summary, these data indicate that, although a higher *CALR*-mutated VAF status is enriched for MF patients with traits of more advanced disease, current findings do not justify the routine implementation of VAF determination in clinical practice, unlike for *JAK2*V617F. Whether a *CALR*-high status has different impact in ET remains to be assessed in future studies.

## Supplementary information

Legend FIGURE S1

FIGURE S1

Supplemental Table 1

## Data Availability

Data not available without request and IRB review due to patient confidentiality.
