# Supplementary material for: Phenotypic correlations of CALR mutation variant allele frequency in patients with myelofibrosis
Source: Blood Cancer J. 2023 Jan 30;13(1):21. doi: 10.1038/s41408-023-00786-x (PMC9884661; doi:10.1038/s41408-023-00786-x)
Supplement: Supplementary file 1 — Legend FIGURE S1 [file 41408_2023_786_MOESM1_ESM.docx]

**Supplement FIGURE LEGEND**

**Figure S1.**

Correlation of *CALR* VAF determination by capillary gel electrophoresis and NGS according to Sperman’s rho test (panel A) and Bland-Altman test (panel B).
