# Supplementary material for: Phenotypic correlations of CALR mutation variant allele frequency in patients with myelofibrosis
Source: Blood Cancer J. 2023 Jan 30;13(1):21. doi: 10.1038/s41408-023-00786-x (PMC9884661; doi:10.1038/s41408-023-00786-x)
Supplement: Supplementary file 2 — FIGURE S1 [file 41408_2023_786_MOESM2_ESM.pptx]

## Slide 1
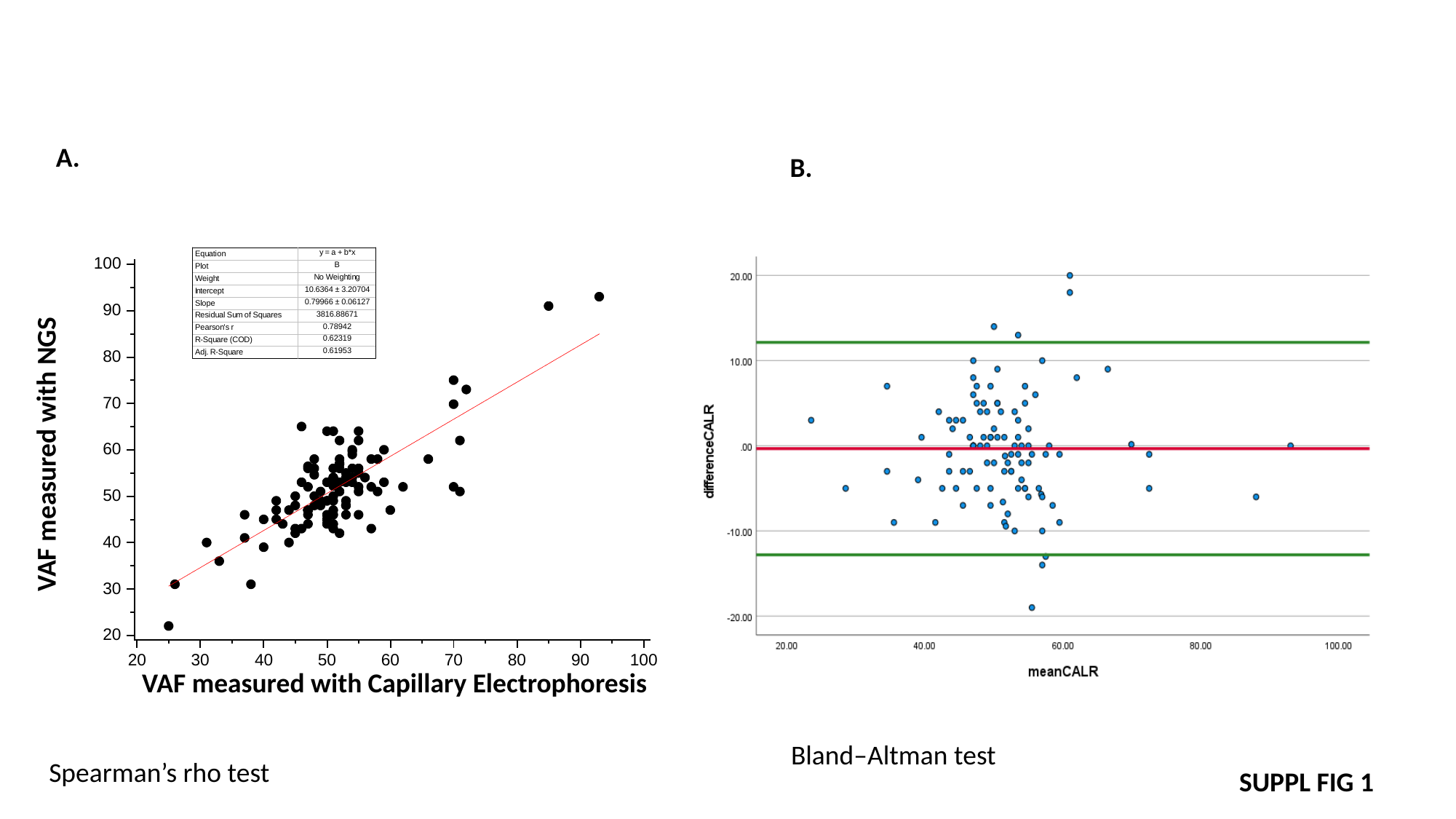

A.
B.
VAF measured with NGS
VAF measured with Capillary Electrophoresis
Bland–Altman test
Spearman’s rho test
SUPPL FIG 1
