# Supplementary material for: Phenotypic correlations of CALR mutation variant allele frequency in patients with myelofibrosis
Source: Blood Cancer J. 2023 Jan 30;13(1):21. doi: 10.1038/s41408-023-00786-x (PMC9884661; doi:10.1038/s41408-023-00786-x)
Supplement: Supplementary file 3 — Supplemental Table 1 [file 41408_2023_786_MOESM3_ESM.docx]

**Supplemental Table 1: Clinical and laboratory characteristics according to diagnosis and *CALR* VAF category (High versus Low).**

| **Variables** | **Prefibrotic-MF (n=42)** | | **P** | **Overt-MF (n=36)** | | | **P** | | **PPV/PET-MF (n=43)** | | | **P** | |
| --- | --- | --- | --- | --- | --- | --- | --- | --- | --- | --- | --- | --- | --- |
|  | ***CALR*-low**  **(N=36)** | ***CALR*-high**  **(N=6)** |  | ***CALR*-low**  **(N=28)** | ***CALR*-high**  **(N=8)** |  | | ***CALR*-low**  **(N=29)** | | ***CALR*-high**  **(N=14)** |  | |  |
| *CALR* mutation type  Type1/1-like  Type2/2-like | 24 (67.7)  12 (33.3) | 4 (67.7)  2 (33.3) | 1.0 | 20 (71.4)  8 (28.6) | 6 (75.0)  2 (25.0) | .84 | | 23 (79.3)  6 (20.7) | | 10 (71.4)  4 (28.6) | .57 | |  |
| Follow-up (yrs); median (range) | 7.0 (0.3-37.1) | 5.2 (2.8-30.1) | .97 | 7.1 (0.3-14.9) | 8.1 (2.5-14.1) | .62 | | 4.3 (0.4-11.4) | | 3.8 (0.6-11.3) | .90 | |  |
| Males; *n* (%) | 15 (41.7) | 4 (66.7) | .25 | 15 (41.7) | 4 (66.7) | .25 | | 15 (41.7) | | 4 (66.7) | .25 | |  |
| Age (yrs); median (range) | 47.1 (18-80) | 40.0 (29-82) | .94 | 61.0 (21-84) | 59.3 (38-65) | .49 | | 59.7 (20-76) | | 64.6 (41-80) | .21 | |  |
| Age >65y; *n* (%) | 8 (22.2) | 1 (16.7) | .76 | 8 (28.6) | 0 (-) | .09 | | 12 (41.4) | | 5 (35.7) | .72 | |  |
| WBC, x 10^9^/L; median (range) | 9.2 (2.5-18.1) | 7.4 (6.9-8.5) | .21 | 6.9 (3.4-56.9) | 11.1 (5.7-22.0) | .22 | | 8.1 (3.3-33.3) | | 6.4 (4.0-36.0) | .14 | |  |
| Hemoglobin, g/L; median (range) | 13.1 (8.0-15.3) | 11.1 (9.5-14.6) | .12 | 12.1 (8.1-14.9) | 10.3 (8.8-14.5) | .26 | | 11.0 (7.4-13.9) | | 10.8 (8.3-14.4) | .98 | |  |
| Platelets, x 10^9^/L; median (range) | 900 (275-1800) | 560 (478-896) | .05 | 447 (59-1500) | 509 (398-666) | .95 | | 434 (57-1506) | | 340 (102-1053) | .36 | |  |
| Circulating blasts ; mean (±SD) | 0.3±1.1 | 0.0±0.0 | .43 | 0.6±1.0 | 1.0±1.0 | .23 | | 2.0±4.1 | | 0.6±1.0 | .23 | |  |
| CD34+x10^6^/L; median (range) | 7.8 (0.7-108) | 74.9 (4.1-146) | .09 | 24.7 (0.0-498) | 225 (18-3432) | **.02** | | 80.3 (0.0-2143) | | 49.1 (4.0-1090) | .40 | |  |
| LDH >UNL; *n* (%)(n=85) | 21 (77.8) | 2 (100) | .45 | 17 (85.0) | 4 (100) | .46 | | 20 (100) | | 12 (100) | 1.0 | |  |
| Splenomegaly; *n* %)(n=115) | 18 (51.4) | 0 (-) | **.02** | 24 (88.9) | 8 (100) | .32 | | 17 (68.0) | | 12 (85.7) | .22 | |  |
| Spleen >10cm from LCM (n=115) | 3 (8.8) | 0 (-) | .07 | 9 (37.5) | 5 (62.5) | .36 | | 13 (52.0) | | 7 (50.0) | .27 | |  |
| Constitutional symptoms; *n* (%)(n=116) | 4 (11.1) | 2 (33.3) | .15 | 8 (29.6) | 3 (37.5) | .67 | | 8 (30.8) | | 5 (38.5) | .63 | |  |
| Karyotype Information; *n* (%) (n=90)  Abnormal cytogenetics  Unfavorable karyotype† | 2 (7.1)  0 (-) | 1 (33.3)  1 (33.3) | .14  .09 | 8 (42.1)  4 (21.1) | 3 (37.5)  1 (12.5) | .82  .53 | | 1 (4.8)  1 (4.8) | | 2 (18.2)  1 (9.1) | .22  .58 | |  |
| IPSS; *n* (%)  Low  Intermediate-1  Intermediate-2  High | 24 (66.7)  8 (22.2)  2 (5.6)  2 (5.6) | 4 (66.7)  1 (16.7)  0 (-)  1 (16.7) | .73 | 11 (39.3)  7 (25.0)  7 (25.0)  3 (10.7) | 3 (37.5)  2 (25.0)  3 (37.5)  0 (-) | .75 | | 8 (27.6)  10 (34.5)  5 (17.2)  6 (20.7) | | 3 (21.4)  6 (42.9)  3 (21.4)  2 (14.3) | .89 | |  |
| MIPSS70/MYSEC-PM; *n* (%)(n=108)  Low  Intermediate(-1)  Intermediate-2  High | 21 (65.6)  6 (18.8)  -  5 (15.6) | 3 (60.0)  1 (20.0)  -  1 (20.0) | .96 | 6 (24.0)  12 (48.0)  -  7 (28.0) | 0 (-)  5 (62.0)  -  3 (37.5) | .41 | | 8 (29.6)  9 (33.3)  6 (22.2)  4 (14.8) | | 5 (45.5)  3 (27.3)  1 (9.1)  2 (18.2) | .69 | |  |
| Total Major Thrombosis events^§^; *n* (%) | 2 (5.6) | 0 (-) | .55 | 3 (10.7) | 2 (25.0) | .30 | | 4 (13.8) | | 0 (-) | .15 | |  |
| Total Major Bleeding events^§^; *n* (%) | 3 (8.3) | 0 (-) | .46 | 7 (25.0) | 0 (-) | .12 | | 4 (13.8) | | 2 (15.4) | .89 | |  |
| Acute Leukemia progression; *n* (%) | 0 (- ) | 0 (-) | nv | 2 (7.1) | 2 (25.0) | .16 | | 5 (17.2) | | 0 (-) | .10 | |  |
| Death; *n* (%) | 6 (16.7) | 1 (16.7) | 1.0 | 11 (39.3) | 5 (62.5) | .24 | | 14 (48.3) | | 6 (42.9) | .74 | |  |
| HMR category; n (%) | 5 (17.7) | 2 (33.3) | .27 | 15 (53.6) | 6 (75.0) | .28 | | 10 (40.0) | | 6 (42.9) | .71 | |  |
| >2 HMR mutated genes | 1 (2.8) | 0 (-) | .37 | 4 (14.3) | 4 (50.0) | .10 | | 3 (10.3) | | 2 (14.3) | .60 | |  |
| Patients with additional mutated myeloid genes; n (%) | 14 (41.2) | 5 (83.3) | **.04** | 19 (67.8) | 8 (100.0) | .06 | | 17 (68.0) | | 10 (71.4) | .82 | |  |

Abbreviations: IPSS, International Prognostic Scoring System. HMR, high molecular risk category, points to the presence of any one mutation in *ASXL1, EZH2, SRSF2, IDH1/2* and *U2AF1*. HMR >2, means the presence of 2 or more HMR mutated genes; 2 or more mutations in the same gene were counted as one. †Unfavorable karyotype indicates any abnormal karyotype other than normal karyotype or sole abnormalities of 20q-, 13q-, +9, chromosome 1 translocation/duplication, -Y or sex chromosome abnormality other than –Y.
